# Beyond dopamine blockade: mechanistic humility and the rise of muscarinic, TAAR1, and glutamatergic pathways in schizophrenia

**DOI:** 10.3389/fpsyt.2026.1769613

**Published:** 2026-04-02

**Authors:** Fawad Taj

**Affiliations:** 1 Department of Psychiatry, University Hospitals Cleveland Medical Center, Cleveland, OH, United States; 2 Case Western Reserve University School of Medicine, Cleveland, OH, United States

**Keywords:** dopamine, muscarinic, schizophrenia, TAAR1, xanomeline–trospium

## Abstract

The approval of the first non–dopamine-blocking therapy for schizophrenia marks a defining moment in psychiatry. Muscarinic M_1_/M_4_ modulation, alongside emerging TAAR1 and glutamatergic pathways, signals a shift beyond dopamine dominance toward circuit-level integration. These advances embody *mechanistic humility*: the scientific courage to prioritize clinical signal over mechanistic certainty. It is the scientific curiosity to revisit older hypotheses, question single-pathway models, and integrate multiple mechanisms. Building on the recognition of dopamine blockade’s experiential burdens, this new era guides psychiatry toward a pluralistic framework. The challenge for 2026 is not to replace dopamine, but to *rebalance* it, moving from receptor blockade dominance to circuit modulation informed pluralistic treatment. This evolution aims to restore harmony not just among neural circuits, but within the lived experience of patients.

## 1 Introduction

The 2024 approval of the dual muscarinic M_1_/M_4_ receptor agonist xanomeline–trospium chloride marks a mechanistic inflection point in schizophrenia treatment. For the first time in decades, antipsychotic efficacy was demonstrated without direct dopamine receptor blockade (1–3). This ends the era of the ‘single therapeutic anchor’. With the expansion of mechanistic options available for schizophrenia, dopamine is now just one pillar in a broader therapeutic architecture.

Dopamine-blocking antipsychotics carry underrecognized costs, including dysphoria, motivational flattening, and diminished reward sensitivity, even without overt extrapyramidal side effects. Marder and Ostlund’s “new conversation with patients” reflects a broader reckoning: symptom control alone has not restored function or quality of life (4).

Recent advances favor integration over replacement. Contemporary models emphasize dysfunction across cortical–striatal–thalamic circuits shaped by interacting cholinergic, glutamatergic, and dopaminergic systems (5–8).

Muscarinic agonism crystallizes this shift. Convergent preclinical, post-mortem, genetic, and neuroimaging evidence implicates muscarinic M_1_ and M_4_ receptors in cortical integration, striatal gating, and cognitive regulation, domains centrally disrupted in schizophrenia (6, 9–13). Early xanomeline trials showed antipsychotic-like effects but were limited by peripheral cholinergic toxicity; trospium co-formulation overcame this barrier and enabled later efficacy without dopamine-associated liabilities (1–3). Beyond symptom control, pooled Phase 3 analyses demonstrate a replicable cognitive benefit in patients with baseline impairment, largely independent of symptom change (14), unlike dopamine-blocking agents (15).

Together, these developments underscore mechanistic humility and a shift toward muscarinic-anchored pluralism in schizophrenia treatment.

## 2 Mechanistic humility

Mechanistic humility is the move away from single-pathway (e.g., the D_2_-only focus), “one-key, one-lock”, pharmacology toward treatments that account brain’s complex, homeostatic circuit balance. It is a discipline of curiosity that requires the scientific courage to prioritize *clinical signal* over *mechanistic elegance*, valuing what works for the patient even before we fully map the circuit. It is grounded in three commitments. First, schizophrenia may respond to interventions acting across interacting circuits rather than a single dominant pathway (5, 7, 8). Second, therapeutic efficacy may precede mechanistic resolution, with clinical benefit emerging before full molecular and circuit explanation (6). Third, models must remain provisional and revisable as outcomes accumulate (6, 13).

Psychiatry has advanced through such asymmetries before: chlorpromazine’s efficacy preceded recognition of dopamine D_2_ blockade as its principal mechanism (6, 16). Operationally, it favors patient-centered outcomes over single-target optimization (5, 13), which means we stop asking ‘Does it block D_2_?’ and start asking ‘Does it restore the function and quality of life?’. It recognizes coordinated cortical–striatal–thalamic modulation (7, 8). Finally, it treats non-response as informative rather than failure of a paradigm, using clinical heterogeneity to refine mechanistic models, identify biological subgroups, and guide future trial design (13, 17).

In drug development, mechanistic humility favors parallel exploration. It raises the bar by demanding integration across scales and responsiveness to clinical reality when mechanistic certainty lags behind clinical benefit (6, 13). New agents are not required to outperform dopamine antagonists; instead, they are evaluated by how they reshape circuit dynamics, experiential burden, and functional capacity (5, 17, 18). This expands the therapeutic design space to muscarinic, TAAR1, and glutamatergic strategies (5, 7, 8, 17).

## 3 Beyond dopamine: interacting circuits

Investigations targeting muscarinic, TAAR1 and glutamatergic systems reinforce that schizophrenia therapeutics is moving toward circuit-level modulation. These approaches differ in maturity and evidentiary depth, yet share a common rationale: reshaping distributed network dynamics. Given the depth of clinical and biological evidence, muscarinic M_1_/M_4_ modulation is examined here as an anchor case, with other mechanisms discussed as complementary rather than equivalent.

### 3.1 Muscarinic agonists

Muscarinic M_1_/M_4_ modulation illustrates how non-dopaminergic mechanisms can influence schizophrenia-relevant circuitry. Rather than targeting a single neurotransmitter output as a ‘mute button’, M_1_ tunes the signal-to-noise ratio in the prefrontal cortex, while M_4_ adjusts the volume in the striatum. Through complementary receptor actions, M_1_/M_4_ agonism reshapes cortical and subcortical processing like a ‘mixing board’.

M_1_ receptors are enriched on pyramidal neurons in the prefrontal cortex and hippocampus, particularly within cortical layers III and V of association cortex (e.g., dorsolateral prefrontal regions), which are critical for cortico-cortical integration and top-down control. Postmortem immunohistochemical studies demonstrate reduced M_1_ receptor–positive pyramidal neurons in these layers in schizophrenia, supporting disease-relevant cortical cholinergic dysfunction (13). Through Gq/11-coupled signaling, M_1_ activation modulates intracellular calcium dynamics and NMDA receptor–linked synaptic plasticity, supporting cortical integration and context-dependent cognition (6, 18–21). In schizophrenia, where cognitive dysfunction is increasingly attributed to impaired prefrontal integration rather than isolated neurotransmitter deficits, M_1_-mediated modulation provides a biologically plausible mechanism for improving cognitive coherence across distributed cortical networks (6, 18, 20).

In contrast, M_4_ receptors are highly expressed in striatal circuitry, particularly on striatal spiny projection neurons and associated interneuron networks, where their activation suppresses dopamine release and modulates corticostriatal signaling. Preclinical studies demonstrate that selective M_4_ activation produces sustained suppression of striatal dopamine output and antipsychotic-like behavioral effects, including normalization of dopaminergic hyperactivity and sensorimotor gating, without direct D_2_ receptor antagonism (10, 22–24). These findings support a circuit-level model in which M_4_ signaling recalibrates striatal gain control and stabilizes cortico–striato–thalamo– cortical loops implicated in aberrant salience assignment.

Together, these distributions support an integrative model in which M_1_ modulates cortical network fidelity and M_4_ recalibrates striatal dopaminergic output within cortico–striato–thalamo–cortical (CSTC) loops, complementing established clinical efficacy and cognitive benefit in human trials (1–3, 14). Critically, the distinction between clinical efficacy and mechanistic explanation must be maintained. Detailed mapping of receptor-level actions onto specific circuit dynamics, including CSTC stabilization and cortical integration, remains a translational inference supported by convergent preclinical and neuroimaging evidence rather than definitive human biomarkers (6, 25). Maintaining this distinction is central to mechanistic humility.

Genetic data provide additional, though more modest, support. CHRM4 variants have been associated with schizophrenia risk and treatment-related phenotypes in some cohorts, suggesting that interindividual differences in M_4_ signaling may influence illness expression or pharmacologic response (26). Although not prominent in large-scale GWAS, these associations align with convergent post-mortem and pharmacologic evidence.

Taken together, these data establish muscarinic M_1_/M_4_ modulation as a biologically grounded anchor for mechanistic integration. The strength of this case lies in the alignment across molecular pathology, cellular architecture, and reproducible clinical efficacy, precisely the conditions under which mechanistic humility is warranted.

### 3.2 TAAR1 agonists

Trace amine–associated receptor 1 (TAAR1) agonism represents a distinct strategy for modulating dopaminergic and glutamatergic signaling without sustained D_2_ receptor antagonism. TAAR1 is expressed in monoaminergic nuclei, including dopaminergic and serotonergic systems, where it functions as a regulator of presynaptic neurotransmitter dynamics rather than a postsynaptic receptor blocker (27). Preclinical models demonstrate that TAAR1 activation reduces dopaminergic neuron firing, modulates striatal dopamine synthesis and release, and influences downstream glutamatergic tone, producing antipsychotic-like effects across behavioral paradigms including hyperlocomotion and prepulse inhibition deficits (28–30). Rather than blocking dopamine transmission, TAAR1 agonists appear to recalibrate its temporal and contextual signaling, aligning with circuit models of aberrant salience.

TAAR1 agonists, such as ulotaront, have demonstrated antipsychotic efficacy in randomized trials (8, 30, 31), with side-effect profile characterized by low extrapyramidal and metabolic liability (29). However, more recent pooled analyses indicate that effect sizes relative to placebo in phase 3 trials have been modest, with substantial placebo response contributing to attenuated separation, and ralmitaront showing lower efficacy than risperidone in direct comparison (31). These findings suggest that while TAAR1 agonism offers a biologically plausible and tolerable alternative mechanism, its magnitude and durability of effect remain under active evaluation.

From a clinical perspective, TAAR1 agonism may be particularly relevant for patients who are sensitive to extrapyramidal or metabolic adverse effects associated with D_2_ receptor antagonists, given its non-D₂-blocking mechanism and relatively favorable tolerability profile in early trials. By modulating presynaptic dopaminergic tone rather than suppressing postsynaptic signaling, TAAR1 agonists may offer a strategy for attenuating psychotic symptoms while preserving physiological dopamine function. However, given the modest effect sizes observed in later-stage studies and the absence of long-term relapse-prevention data, TAAR1 agonists are best conceptualized at present as emerging alternatives whose optimal role, first-line, adjunctive, or mechanism-guided switch, remains to be defined through further comparative trials.

### 3.3 Glutamatergic strategies

Glutamatergic hypotheses of schizophrenia are grounded in evidence that NMDA receptor hypofunction can reproduce key features of psychosis, cognitive fragmentation, and impaired cortical integration. Preclinical models link NMDA disruption to altered excitation–inhibition balance, interneuron dysfunction, and impaired oscillatory synchrony, processes central to cognitive coherence (5, 7). Pharmacologic strategies aimed at augmenting glutamatergic signaling have yielded mixed clinical results. These outcomes are increasingly interpreted as evidence of biological heterogeneity and challenges in target engagement and trial design (5, 7). Within an integrative framework, glutamatergic agents are best understood as upstream circuit stabilizers that may preferentially influence cognition and negative symptoms rather than positive symptom suppression.

### 3.4 Other emerging directions

α_5_-GABA_a_ receptor potentiators are motivated by evidence that impaired inhibitory control contributes to network noise in schizophrenia, particularly within hippocampal– prefrontal circuits; clinical development remains early and evidence for standalone antipsychotic efficacy is limited (32).

## 4 Therapeutic efficacy

### 4.1 Clinical evidence for muscarinic M/M modulation

The antipsychotic efficacy of muscarinic M_1_/M_4_ modulation (xanomeline–trospium chloride/KarXT) is established across multiple randomized, double-blind, placebo controlled trials in acutely psychotic patients with schizophrenia (33). In the Phase 3 EMERGENT-3 trial, KarXT produced a placebo-adjusted reduction of 8.4 points in PANSS total score at week 5 (Cohen’s *d* ≈ 0.60), a magnitude comparable to that observed with commonly used dopamine-blocking antipsychotics in acute studies (1). Additionally, 50.6% of patients treated with KarXT achieved a ≥30% reduction in PANSS total score, compared with 25.3% of placebo-treated patients, representing a clinically meaningful separation (1). A Bayesian meta-analysis pooling three randomized controlled trials (n = 674) reported a mean PANSS total improvement of approximately 9.7 points versus placebo, with significant effects across both positive and negative symptom domains and no increase in serious adverse events (34). Clinician-rated global severity also improved significantly, supporting the clinical relevance of symptom change (1).

KarXT’s tolerability profile differs qualitatively from that of dopamine antagonists and is central to its clinical interpretation. The most common treatment-emergent adverse events are cholinergic, primarily gastrointestinal, including nausea, vomiting, and constipation; these effects are typically early in onset, mild-to-moderate, and transient, with discontinuation rates comparable to placebo (1, 34). Importantly, KarXT does not carry intrinsic risks of extrapyramidal symptoms, prolactin elevation, or clinically meaningful weight gain (1, 34).

From a clinical standpoint, KarXT shifts adherence management from cumulative metabolic and motor toxicity toward early tolerability optimization, emphasizing anticipatory counseling, gastrointestinal symptom management, and dose adjustment during the initial treatment window.

Cognitive impairment remains a core determinant of functional outcome in schizophrenia and has historically shown minimal response to dopamine-blocking antipsychotics (15). In contrast, pooled analyses from two Phase 3 trials demonstrate a replicable cognitive benefit with KarXT in patients with baseline impairment, with medium-to-large effect sizes that are independent of changes in positive or negative symptoms (14). While these findings do not establish universal pro-cognitive efficacy, they represent a meaningful departure from prior pharmacologic experience.

Important evidence gaps remain. The pivotal EMERGENT trials were of short duration (~5 weeks) and were not designed to assess relapse prevention or long-term functional outcomes (1). In the 52-week, open-label EMERGENT-5 trial (35), psychiatrically stable adults with schizophrenia were safely switched from prior antipsychotics to xanomeline–trospium, with sustained improvement in PANSS total, positive, and negative scores and no signal of symptom worsening over one year. Treatment was generally well tolerated, with adverse events consistent with prior short-term trials and no emergence of extrapyramidal symptoms, hyperprolactinemia, or clinically meaningful metabolic deterioration. Notably, mean body weight decreased over 52 weeks, and movement disorder scales showed slight improvement. While the open-label design and lack of a comparator arm preclude definitive conclusions regarding relapse prevention or comparative maintenance efficacy, EMERGENT-5 provides important evidence that muscarinic M_1_/M_4_ modulation can support long-term symptom stability following discontinuation of dopamine-blocking antipsychotics.

Comparative evidence indicates that KarXT and commonly used dopamine-blocking antipsychotics are all more effective than placebo in acute schizophrenia, with no clear short-term superiority of KarXT (36). The paradigm shift represented by KarXT therefore lies not in superior acute efficacy, but in achieving antipsychotic benefit while reconfiguring the long-term tolerability landscape (36, 37).

### 4.2 Therapeutic implications and clinical translation

The distinction between integration and replacement has direct implications for clinical practice. If mechanistic humility requires taking reproducible clinical benefit seriously, it also requires reconsidering long-standing treatment hierarchies. As emphasized by Marder and Ostlund’s call for a “new conversation with patients,” shared decision-making should center not only on symptom control but also on the experiential, cognitive, and functional costs associated with dopamine blockade (4). That conversation is incomplete if treatments with comparable efficacy and a fundamentally different adverse-effect profile are systematically deferred.

If the dopamine era emphasized control, the muscarinic era invites choice. Introducing KarXT earlier in the treatment algorithm or as a first-line *consideration* in selected patients operationalizes Marder and Ostlund’s call (4). To treat KarXT merely as a ‘backup’ for dopamine failure is to misinterpret its efficacy, tolerability profile, and emerging durability data. For patients prioritizing cognition and metabolic health, it is reasonable first-choice for schizophrenia, while the evidence base continues to mature.

First, KarXT demonstrates clinically meaningful reductions in PANSS total and positive symptoms, across Phase 2 & 3 trials, with response rates comparable to dopamine-blocking antipsychotics (1, 34). Secondly, it does not carry intrinsic risks of extrapyramidal symptoms, prolactin elevation, or weight gain; adverse effects that influence adherence and quality of life (1, 34, 36). Cholinergic adverse effects are common but typically early, mild-to-moderate, and manageable, with discontinuation rates similar to placebo (1, 34). Although randomized relapse-prevention trials remain necessary, 52-week open-label data demonstrate sustained symptom improvement and tolerability over one year of continuous KarXT treatment (35). This trade-off is particularly relevant early in illness when initial treatment experiences shape long-term engagement (38). Finally, cognitive impairment, a core determinant of functional outcome in schizophrenia, shows minimal response to dopamine-blocking antipsychotics (15). In contrast, pooled analyses from Phase 3 trials demonstrate a replicable cognitive benefit with KarXT in patients with baseline impairment, with medium-to-large effect sizes that are independent of changes in positive or negative symptoms (14).

A first-line posture toward KarXT does not imply exclusivity. Mechanistic humility cautions against both premature combination and reflexive reliance on a single mechanism (6). Dopamine antagonists remain essential tools, particularly for agitation and relapse prevention. However, mechanistic integration suggests that sequencing and selective combination, rather than rigid hierarchies, may ultimately define best practice.

## 5 Discussion and conclusion

For decades, dopamine D_2_ receptor blockade has anchored schizophrenia treatment. Its effectiveness in reducing psychosis reshaped psychiatric care and provided long-needed stability. Yet clinical experience has also revealed its limitations: persistent cognitive impairment, motivational blunting, metabolic burden, and incomplete functional recovery for many patients (4, 15, 37). These trade-offs have often been accepted as unavoidable.

The emergence of muscarinic agonism challenges that assumption. Xanomeline–trospium demonstrates that antipsychotic efficacy can be achieved without direct dopamine blockade, while reshaping the adverse-effect landscape and offering the possibility of cognitive benefit in a subset of patients (1, 14, 34, 35). This does not invalidate dopamine-based models; rather, it reframes them as necessary but not sufficient.

Psychopharmacology has advanced through similar moments before. Clozapine’s broad receptor engagement, once dismissed as “dirty pharmacology”, proved essential for treatment-resistant schizophrenia, illustrating that clinical efficacy can precede mechanistic clarity (16, 37). Muscarinic modulation represents a contemporary expression of this principle.

Mechanistic humility, as articulated here, is therefore not a retreat from rigor but an advance in it. It recognizes schizophrenia as a disorder of distributed circuit dysfunction, accepts that multiple biological levers can yield antipsychotic signal, and allows clinical outcomes to guide refinement of mechanistic understanding. The convergence of postmortem pathology, circuit-level hypotheses, and reproducible clinical efficacy positions muscarinic M_1_/M_4_ modulation as a credible anchor for integrative therapeutics (6, 11–13).

Clinically, this shifts first-line conversations toward mechanism-informed choice (4). Offering KarXT as a first-line option operationalizes the “new conversation with patients” proposed by Marder and Ostlund, one that weighs symptom control alongside cognition, tolerability, and long-term functioning (4). It enables choice between mechanisms, not just molecules, while remaining responsive to evolving evidence.

Important questions remain, including relapse prevention, biomarker-guided stratification, and real-world functional outcomes. These gaps, however, do not diminish what has already been demonstrated. Importantly, mechanistic humility does not preclude clinical judgment or early adoption, but frames such decisions as provisional, patient-specific, and responsive to emerging evidence.

The dopamine era offered control; muscarinic modulation expands capacity for individualized, circuit-informed care. We are moving from silencing the symptoms to strengthening the circuits that allow the self to emerge. Mechanistic humility provides a framework to navigate this transition, integrating efficacy with patient experience and emerging evidence. Mechanistic humility is the wisdom to accept that while we may not yet have the full map of the mind, we have the tools to help patients navigate it.

## Data Availability

The original contributions presented in the study are included in the article/supplementary material. Further inquiries can be directed to the corresponding author.
